# Psycho-Covid

**DOI:** 10.1192/j.eurpsy.2022.1353

**Published:** 2022-09-01

**Authors:** M.J. Gordillo Montaño, S.V. Boned Torres, L. Rodriguez

**Affiliations:** Hospital Can Misses, Psychiatry, Eivissa, Spain

**Keywords:** stressor, Covid-19, Psychotic disorder, pandemic

## Abstract

**Introduction:**

The COVID-19 pandemic generated a health emergency and led to the adoption of different measures, including home quarantine and social isolation, which, as we have seen, has had an impact on the mental health of the majority of citizens, with the possibility of psychiatric disorders appearing. in people without prior mental illness, such as acute decompensations in patients with known disorders, more vulnerable to environmental stressors.

**Objectives:**

Learn and rethink alarm signals in extreme situations such as the one experienced in recent months, as well as observe the impact, negative in many cases, but positive in others, of the patients we treat daily.

**Methods:**

Description through brief clinical cases of the impact of the COVID-19 pandemic on psychotic patients and the decompensation that it has entailed, including due to confinement measures and social isolation, associated with over-information through the media, chaos initial and the uncertainty that it caused and the associated fear.

**Results:**

Restrictions as a result of COVID-19 have played a very relevant role as an external stressor for the appearance of psychopathological alterations, including psychotic symptoms. In addition, people who suffer from psychosis or at risk of psychotic disorder can be especially affected and trigger acute psychopathology with social isolation, loss of daily routines, unemployment, homelessness.

**Conclusions:**

These cases are an example that shows the need for an early and effective approach to the rise in mental illnesses in circumstances of this caliber.

**Disclosure:**

No significant relationships.

